# Efficient Solar‐Driven CO_2_ Methanation and Hydrogen Storage Over Nickel Catalyst Derived from Metal–Organic Frameworks with Rich Oxygen Vacancies

**DOI:** 10.1002/advs.202304406

**Published:** 2023-10-22

**Authors:** Huiling Wang, Qiang Li, Jin Chen, Jing Chen, Hongpeng Jia

**Affiliations:** ^1^ Xiamen Key Laboratory of Materials for Gaseous Pollutant Control Institute of Urban Environment Chinese Academy of Sciences Xiamen 361021 China; ^2^ Key Laboratory of Urban Pollutant Conversion Institute of Urban Environment Chinese Academy of Sciences Xiamen 361021 China; ^3^ College of Life Science Fujian Agriculture and Forestry University Fuzhou 350002 China; ^4^ University of Chinese Academy of Sciences Beijing 100049 China; ^5^ Fujian Institute of Research on the Structure of Matter Chinese Academy of Sciences Fuzhou 350002 China

**Keywords:** hydrogen storage, metal–organic frameworks, nickel catalyst, photothermal catalysis, solar‐driven CO_2_ methanation

## Abstract

Solar‐driven photothermal conversion of carbon dioxide (CO_2_) to methane (CH_4_) is a promising approach to remedy energy shortage and climate changes, where highly efficient photothermal catalysts for CO_2_ methanation urgently need to be designed. Herein, nickel‐based catalysts (Ni/ZrO_2_) derived from metal–organic frameworks (MOFs) are fabricated and studied for photothermal CO_2_ methanation. The optimized catalyst 50Ni/ZrO_2_ achieves a stable CH_4_ production rate of 583.3 mmol g^−1^ h^−1^ in a continuous stability test, which is almost tenfold higher than that of 50Ni/C‐ZrO_2_ synthesized via commercial ZrO_2_. Physicochemical properties indicate that 50Ni/ZrO_2_ generates more tetragonal ZrO_2_ and possesses more oxygen vacancies (OVs) as well as enhanced nickel‐ZrO_2_ interaction. As a result, 50Ni/ZrO_2_ exhibits the strong abilities of light absorption and light‐to‐heat conversion, superior adsorption capacities of reactants (H_2_, CO_2_), and an intermediate product (CO), which finally boosts CH_4_ formation. This work provides an efficient strategy to design a photothermocatalyst of CO_2_ methanation through utilizing MOFs‐derived support.

## Graphical abstract

Solar‐driven photothermal carbon dioxide (CO_2_) methanation over the 50Ni/ZrO_2_ derived from metal–organic frameworks (MOFs) achieves a CH_4_ production rate of 583.3 mmol g^−1^ h^−1^ which is tenfold higher than that of 50Ni/C‐ZrO_2_. The effective light‐to‐heat conversion and more OVs jointly promote the CO_2_ methanation and hydrogen storage. The formation of key intermediates indicates the possible reaction pathways over 50Ni/ZrO_2_.

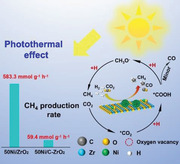

## 1 Introduction

The excessive emission of carbon dioxide (CO_2_) causes energy crises and environmental problems.^[^ ^1^, ^2^ ^]^ CO_2_ methanation is an attractive and environment‐friendly technology to convert CO_2_ into value‐added fuels and simultaneously offers a way of storing H_2_.^[^ ^3^, ^4^ ^]^ Commonly, thermal catalytic technology is an alternative method to achieve highly efficient CO_2_ conversion. However, CO_2_ molecules possess extraordinary thermodynamic stability and the methanation process is an exothermic reaction (CO_2_ + 4H_2_ ↔ CH_4_ + 2H_2_O (g), Δ*H*_298K_ = −164.9 kJ mol^−1^).^[^ ^5^, ^6^ ^]^ Hence, thermal CO_2_ methanation requires a relatively high temperature or ultrahigh‐pressure in general, resulting in excess energy consumption. The photothermal effect, which converts solar energy into heat, can promote the catalytic reaction. Therefore, photothermal catalysis has attracted tremendous interest from academia and industry due to the efficient utilization of renewable solar energy for CO_2_ methanation as a green technology.^[^ ^7^, ^8^, ^9^ ^]^

In the case of photothermal catalytic reactions, many catalysts have been exploited. For instance, the noble metal catalysts (Ru, Pd, Rh, etc.) have been applied to CO_2_ methanation, but their expensive nature hinders their development and practical applicability.^[^ ^10^, ^11^, ^12^ ^]^ Some transition metals (Ni, Co, Fe, etc.) have also been investigated as alternative catalysts for CO_2_ methanation and show better photothermal conversion efficiency and catalytic performance.^[^ ^13^, ^14^, ^15^, ^16^ ^]^ Among them, the Ni‐based catalysts are considered ideal catalytic materials for CO_2_ methanation owing to their competitive activity, high methane selectivity, good stability, and low cost.^[^ ^17^, ^18^, ^19^, ^20^, ^21^ ^]^ The previous literature reports that Ni/Al_2_O_3_ shows better CO_2_ reduction catalytic activity than precious metals under solar irradiation,^[^ ^22^ ^]^ and Ni‐Al_2_O_3_/SiO_2_ exhibits higher methane yield than Ru/SiO_2_ under full light spectrum irradiation.^[^ ^23^, ^24^ ^]^ The photothermal catalytic production rate of CH_4_ reaches 488 mmol g^−1^ h^−1^ over the metal–organic frameworks (MOFs) derived Ni‐based catalyst under UV–vis–IR irradiation.^[^ ^25^ ^]^ In view of the high performance, abundance, and inexpensiveness of nickel, the catalyst that contains a higher Ni loading, of course, is a priority goal. Indeed, the activity of catalysts not only relies on metallic nanoparticles (NPs) but also relates to the structure of supports. The supported metal catalysts contribute to high metal NPs dispersion, better CO_2_ adsorption, and superior conversion.^[^ ^26^, ^27^ ^]^ MOFs have been widely applied in heterogeneous catalysis due to their high specific surface areas, adjustable band structure, and porous structure characteristics.^[^ ^28^, ^29^ ^]^ The metal NPs supported on MOFs pyrolytic metal oxides possess small size and good dispersion, thus favoring the catalytic performance in catalytic reactions.^[^ ^30^ ^]^

In this work, we took advantage of MOF pyrolytic metal oxides to prepare a series of Ni‐based catalysts. The optimized catalyst 50Ni/ZrO_2_ showed a fine photothermal performance during light‐driven CO_2_ hydrogenation. The CH_4_ production rate reached 583.3 mmol g^−1^ h^−1^, which was almost tenfold higher than that of 50Ni/C‐ZrO_2_ synthesized via commercial ZrO_2_ under full spectrum irradiation. In the follow‐up stability experiment, no nanoparticle aggregation or significant inactivation was observed over 50Ni/ZrO_2_. And physicochemical properties of 50Ni/ZrO_2_ were further investigated and demonstrated that strong light absorption, highly efficient light‐to‐heat conversion, good adsorption capacity of reactants, and sufficient OVs account for the enhanced photothermal catalytic activity. In this study, 50Ni/ZrO_2_ with better catalytic performance and cost advantage suggests potential application in photothermal CO_2_ methanation and meanwhile provides an idea for constructing supported photothermal catalysts.

## 2 Results and Discussion

### 2.1 Structural and Morphological Characterizations

**Figure 1:**
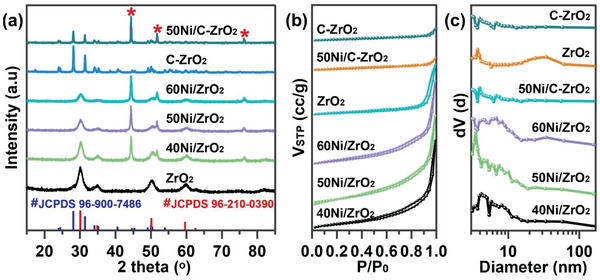
a) XRD patterns. b) N_2_ adsorption‐desorption isotherms. c) Pore distribution of samples.

The crystal structure of synthesized materials was investigated by X‐ray diffraction (XRD), as shown in **Figure** **1**a. The diffraction peaks of C‐ZrO_2_ and 50Ni/C‐ZrO_2_ are matched with monoclinic ZrO_2_ crystal phase (JCPDS 96‐900‐7486, m‐ZrO_2_), which indexes at 2*θ* = 24.1^o^, 28.2^o^, 31.5^o^, 34.2^o^, 35.3^o^, 49.3^o^, and 50.1^o^. While the diffraction peaks at 2*θ* = 30.3^o^, 35.4^o^, 50.4^o^, 59.5^o^, and 73.2^o^ of xNi/ZrO_2_ samples are assigned to tetragonal ZrO_2_ crystal phase (JCPDS 96‐210‐0390, t‐ZrO_2_). It has been previously reported that the existence of a tetragonal ZrO_2_ phase is helpful in achieving higher photocatalytic performance of CO_2_ methanation due to the large amount of CO_2_ adsorption sites over tetragonal ZrO_2_.^[^ ^35^ ^]^ The main characteristic peaks of Ni (111), Ni (200), and Ni (220) lattice planes located at 44.5^o^, 51.8^o^, and 76.4° can be clearly identified over the Ni‐based catalysts, respectively. It illustrates that Ni NPs have been successfully impregnated. The XRD patterns of Ni‐based catalysts display that the peaks of tetragonal ZrO_2_ dwindle relatively with the increasing Ni content. It suggests that the loading of Ni can influence the crystallization process of ZrO_2_ during the pyrolysis of UiO‐66.

The properties of catalysts have been listed in **Table** **1**. The exact content of Ni NPs in the catalysts was analyzed by ICP‐OES. The specific surface areas and pore size distribution curves of all samples were calculated by BET and BJH methods, respectively, based on the nitrogen static adsorption‐desorption isotherms (Figure 1b,c, Figure S1, Supporting Information). The *S*_BET_, *D*_BJH,_ and total pore volume of UiO‐66 are 1241.0 m^2^ g^−1^, 3.0 nm, and 0.7 cm^3^ g^−1^, respectively. The large pore size and total pore volume indicate that UiO‐66 has developed a pore structure, which is conducive to the rapid diffusion of Ni ions and homogeneous dispersion within the interior of the UiO‐66. Moreover, the large S_BET_ provides sufficient sites for the adsorption of Ni ions and is beneficial to the distribution of NiO on the 50NiO/ZrO_2_ surface. By the way, this pore structure of UiO‐66 may restrict the growth of Ni particles and avoid their agglomeration due to the limiting effect during the calcination process. The *S*_BET_ and pore size are closely related to the loading content of Ni over the catalysts. The *S*_BET_ of pure ZrO_2_ is 36.8 m^2^ g^−1^, however, the S_BET_ of xNi/ZrO_2_ (*x* = 40, 50, and 60) increases to 94.1, 122.0, and 90.7 m^2^ g^−1^, respectively, indicating the introduction of Ni obviously improves the *S*_BET_ and pore volume of catalysts. In general, the higher surface area is beneficial for the catalytic activity of CO_2_ methanation.

**Table 1:** Element composition and properties of samples.

Catalysts	Ni loading [wt.%]^a)^	S_BET_ [m^2^ g^−1^]	D_BJH_ [nm]	Ni NPs Size [nm]^b)^	Total pore volume [cm^3^ g^−1^]
UiO‐66	0	1241	3.0	–	0.70
ZrO_2_	0	36.8	3.7		0.23
C‐ZrO_2_	0	15.9	3.0	–	0.06
40Ni/ZrO_2_	40.7	94.1	4.2	34.3	0.42
50Ni/ZrO_2_	50.8	122.0	3.5	33.6	0.42
60Ni/ZrO_2_	61.6	90.7	6.8	34.8	0.39
50Ni/C‐ZrO_2_	50.2	18.1	3.9	42.5	0.06

^a)^ Ni content in the catalysts was analyzed by ICP‐OES;

^b)^ Determined on the basis of TEM images.

**Figure 2:**
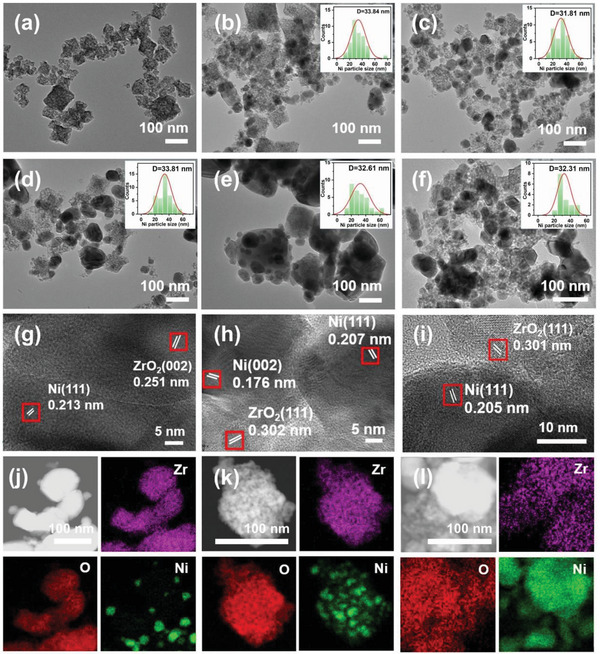
a–f) TEM images of ZrO_2_, 40Ni/ZrO_2_, 50Ni/ZrO_2_, 60Ni/ZrO_2_, 50Ni/C‐ZrO_2_, 50Ni/ZrO_2_‐used. g–i) HRTEM images of 50Ni/C‐ZrO_2_, 50Ni/ZrO_2_, 50Ni/ZrO_2_‐used. j–l) Elemental distribution spectra (EDS) of 50Ni/C‐ZrO_2_, 50Ni/ZrO_2_, and 50Ni/ZrO_2_‐used, respectively.

The morphology of the catalysts was studied by TEM and HRTEM, as shown in **Figure** **2**. Pure ZrO_2_ displays the square shape in different sizes (Figure 2a). Notably, the Ni NPs over fresh and used 50Ni/ZrO_2_ are more uniform compared with those of 40Ni/ZrO_2_, 60Ni/ZrO_2_, and 50Ni/C‐ZrO_2_ (Figure 2b–f). Representative HRTEM images are displayed in Figure 2g–i. The HRTEM image of 50Ni/ZrO_2_ displays the lattice fringe of 0.302 and 0.207 nm, corresponding to the (111) facet of ZrO_2_ and the (111) plane of Ni nanocrystals, respectively. The sizes of Ni NPs over Ni‐based samples estimated from TEM images are listed in Table 1. The Ni NPs size of 50Ni/ZrO_2_ is ≈31.8 nm, which is slightly smaller than that of 50Ni/C‐ZrO_2_ (≈32.6 nm). In addition, the Ni NPs size of used 50Ni/ZrO_2_ which shows almost no change is ≈32.3 nm after the photothermal reduction. It reveals that the Ni NPs of the used 50Ni/ZrO_2_ do not agglomerate during the CO_2_ methanation process. The element mapping images of fresh and used 50Ni/ZrO_2_ (Figure 2k,l) further verify the Ni element distribution is more dispersed after the CO_2_ methanation reaction, possibly due to the influence of the reaction atmosphere on the active site and results in the change of the Ni particle dispersion. Moreover, Ni NPs over 50Ni/ZrO_2_ are more evenly distributed compared to the 50Ni/C‐ZrO_2_ (Figure 2j).

### 2.2 Photothermal Catalytic Performance of CO_2_ Methanation

**Figure 3:**
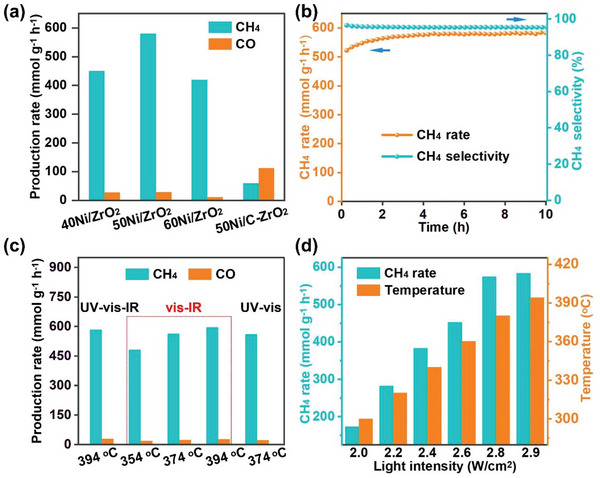
a) Production rate in the initial 5 h of 40Ni/ZrO_2_, 50Ni/ZrO_2_, 60Ni/ZrO_2_, and 50Ni/C‐ZrO_2_ under light intensity of 2.9 W cm^−2^. b) Durability test of 50Ni/ZrO_2_. c) CH_4_ formation rate under different light irradiation of 50Ni/ZrO_2_. d) CH_4_ formation rate and equilibrium temperature of 50Ni/ ZrO_2_ under different light intensity. The catalytic reaction was performed under the focus light via a Fresnel lens in a continuous flow of 10 vol.% CO_2_, 40 vol.% H_2_, and 50 vol.% He (25 mL min^−1^). The vis–IR and UV–vis light were obtained by using the different cutoff filters.

Light‐driven photothermal CO_2_ methanation reaction was performed in a stainless‐steel reactor with a quartz window. A constant mixed gas of 10%CO_2_/40%H_2_/50%He was introduced into the reactor with a rate of 25 mL min^−1^. During the 5 h photothermal CO_2_ reduction test, no products can be detected over pristine ZrO_2_ (Figure S2, Supporting Information), which indicates that neither photocatalytic nor photothermal catalytic activity occurs over pure ZrO_2_ support. An activity evaluation experiment was performed over the 50Ni/ZrO_2_ and 50Ni/ C‐ZrO_2_ under 5%H_2_/Ar atmosphere to eliminate the interference of carbon substance at the same time. As shown in Figures S2 and S3 (Supporting Information), no products can be detected over the 50Ni/ZrO_2_ and 50Ni/ C‐ZrO_2_, which indicates that the carbon‐containing products obtained in CO_2_ methanation were all derived from CO_2_ in feed gas (10%CO_2_/40%H_2_/50%He) during CO_2_ methanation. 50Ni/C‐ZrO_2_ shows *r*_CH4_ of 59.4 mmol g^−1^ h^−1^ and poor CH_4_ selectivity (*S*_CH4_) of ≈35%, as displayed in **Figure** **3**a. Nevertheless, the synthesized MOF‐derived xNi/ZrO_2_ catalysts exhibit significantly enhanced photothermal CO_2_ methanation, among which 50Ni/ZrO_2_ demonstrates the optimal *r*_CH4_ and *r*_CO_ of 583.3 and 28.4 mmol g^−1^ h^−1^, respectively. Consequently, *r*_CH4_ and *S*_CH4_ (95%) of 50Ni/ZrO_2_ are ≈10 and ≈2.7 times higher than those of 50Ni/C‐ZrO_2_, respectively. Catalytic replication experiments are performed to study the selectivity, stability, and reproducibility of the catalyst in CO_2_ reduction. Herein, as shown in Figure S4 (Supporting Information), the *r*_CH4_ was obtained for three replication experiments of photothermocatalytic performance over 50Ni/ZrO_2_ under irradiation, which exhibit stable catalytic activity with a value of more than 560 mmol g^−1^ h^−1^ and selective of 96% in the initial 2 h for each experiment. Furthermore, the production rate of 50Ni/ZrO_2_ maintains stable during 10 h CO_2_ methanation (Figure 3b) with high *S*_CH4_ (95%). Notably, the catalytic activity increased slightly in the early stage of the reaction process, which may be related to the change in the dispersion of Ni particles (Figure 2k,l). The XRD pattern (Figure S5, Supporting Information) of the used 50Ni/ZrO_2_ shows no obvious change compared to the fresh catalyst, together confirming the structure stability of 50Ni/ZrO_2_ during the reaction. Moreover, 50Ni/ZrO_2_ in this work exhibits a considerable catalytic performance compared to the recently reported catalysts for photothermal CO_2_ hydrogenation (Table S1, Supporting Information). Therefore, 50Ni/ZrO_2_ derived from UiO‐66 is confirmed to be a preferential catalyst of high activity, selectivity, and stability for photothermal CO_2_ methanation.

To further investigate the influence of different light regions on the photothermal CO_2_ methanation, the catalytic performance of 50Ni/ZrO_2_ was evaluated under controlled conditions (Figure 3c). After cutting off the UV light, the surface temperature of the catalyst reduces to 354 °C. Subsequently, *r_CH4_* falls to 481.4 mmol g^−1^ h^−1^ under vis–IR light irradiation. However, after heightening incident light intensity to reach the similar temperature of full spectrum (394 °C), *r*_CH4_ dramatically enhances to an identical value of 595.6 mmol g^−1^ h^−1^. Moreover, when cutting off the IR light and tuning light intensity with the temperature of 374 °C, *r*_CH4_ exhibits a parallel value (560.1 mmol g^−1^ h^−1^) under UV–vis light in comparison to that under vis–IR light (562.4 mmol g^−1^ h^−1^). Therefore, it can be found that 50Ni/ZrO_2_ presents similar catalytic activity under different light irradiation when the temperature reaches identical values. We further performed the photothermal CO_2_ catalytic evolution over 50Ni/ZrO_2_ under different light intensities. As displayed in Figure 3d, the surface temperature and *r*_CH4_ of 50Ni/ZrO_2_ enhance proportionally with the increasing light intensity. This evidently demonstrates the reaction temperature plays a crucial role in the photothermal catalytic process, while the traditional photocatalysis involving light‐induced electrons shows no effect in the current system.^[^ ^11^ ^]^ The result is consistent with the thermal catalytic performance of CO_2_ methanation that 50Ni/ZrO_2_ exhibits a similar *r*_CH4_ during light‐driven and thermal‐driven catalysis when under a similar surface temperature (Figure S6, Supporting Information). In conclusion, the photothermal CO_2_ methanation over the 50Ni/ZrO_2_ in this work is intrinsically a light‐driven thermal catalysis, and light which only serves as a heat‐energy supplier is converted to high temperature over the catalyst to trigger the CO_2_ methanation process.

### 2.3 The Origin of Enhanced Photothermal Catalytic Performance

**Figure 4:**
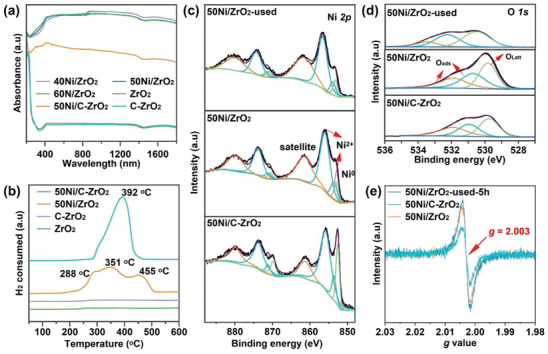
a) DRS spectra of samples. b) H_2_‐TPR profiles of catalysts. c‐d) High‐resolution XPS data of Ni 2p and O 1s collected from as‐synthesized 50Ni/ZrO_2_, 50Ni/C‐ZrO_2_, and 50Ni/ZrO_2_‐used. e) EPR spectra of 50Ni/ZrO_2_, 50Ni/C‐ZrO_2_, and 50Ni/ZrO_2_‐used‐5h.

To figure out the reason why 50Ni/ZrO_2_ exhibits superior CO_2_ methanation performance to 50Ni/C‐ZrO_2_, we first measured the light absorption spectra of the catalysts in the full spectrum, as shown in **Figure** **4**a. Both ZrO_2_ and C‐ZrO_2_ show poor light absorption in the range of 200–2000 nm. Nevertheless, the Ni‐based catalysts exhibit much higher light absorption. More importantly, xNi/ZrO_2_ catalysts possess a much stronger signal response than 50Ni/C‐ZrO_2_ in the whole region, verifying the xNi/ZrO_2_ can contribute to high light absorption capacity. The stronger capacity of light absorption can realize an intensive photothermal effect over the xNi/ZrO_2_ catalysts, as confirmed by the monitored surface temperatures of catalysts (Figure S7, Supporting Information) during the reaction process. The central temperatures of 40Ni/ZrO_2_, 50Ni/ZrO_2_, and 60Ni/ZrO_2_ catalysts arrive at 382, 394, and 391 °C, respectively, which are much higher than those of 50Ni/C‐ZrO_2_ (324 °C) and pure ZrO_2_ (155 °C). Efficient capacity of light‐to‐heat which makes the center temperature of catalyst upgrade a desirable level to promote the photothermal catalysis. Hence, the high light‐induced temperature over the xNi/ZrO_2_ is favorable for the CO_2_ methanation reaction. The H_2_‐temperature‐programmed reduction (H_2_–TPR) profiles of the unreduced samples (50NiO/ZrO_2_ and 50NiO/C‐ZrO_2_) and ZrO_2_ supports are presented in Figure 4b. Both ZrO_2_ and C‐ZrO_2_ seldom consume H_2_ in the range of 50–600 °C, while 50NiO/ZrO_2_ and 50NiO/C‐ZrO_2_ show two parts of reduction areas located in the low‐ and high‐temperature regions (200‐400 and 400–500 °C, respectively). Apparently, the main peak of 50NiO/C‐ZrO_2_ is located at 392 °C assigned to low‐temperature regions. However, 50NiO/ZrO_2_ exhibits peaks not merely at low temperatures of 288 and 351 °C, but a largely composed peak at 455 °C corresponding to high‐temperature regions. As reported, the reduction in the low‐temperature region is mainly attributed to free NiO particles that weakly interact with the support.^[^ ^19^ ^]^ Whereas the high‐temperature peaks are assigned to the reduction of NiO species interacting strongly with the ZrO_2_, which can be reduced to active metallic Ni and is beneficial for CO_2_ methanation.^[^ ^36^ ^]^ On the other hand, the reduction of the NiO species causes different amounts of defects that provide the anchoring sites for Ni NPs and is related to the interaction strength with the support.^[^ ^36^, ^37^ ^]^ Therefore, a stronger interaction may occur between Ni species and ZrO_2_ support in 50Ni/ZrO_2_ which is more favorable to the CO_2_ hydrogenation compared with 50Ni/C‐ZrO_2_.^[^ ^38^ ^]^ The surface chemical states of 50Ni/C‐ZrO_2_ and 50Ni/ZrO_2_ are investigated by X‐ray photoelectron spectroscopy (XPS). The Ni 2p3/2 profiles (Figure 4c) of 50Ni/C‐ZrO_2_ show the peak at (852.6 eV) assigned to Ni^0^. The peaks at 853.6 and 855.8 eV belong to Ni^2+^ in the nickel‐oxide phase. The one at 861.2 eV is a satellite peak from Ni^2+^, which resulted from the oxidation by air during sample transportation.^[^ ^39^, ^40^, ^41^ ^]^ As for 50Ni/ZrO_2_, due to the presence of NiO species which is more difficult to reduce during the hydrogen reduction process as confirmed by H_2_‐TPR profiles (Figure 4b), the Ni^0^ peak is weaker than that of 50Ni/C‐ZrO_2_; however, the binding energy of Ni^0^ over 50Ni/ZrO_2_ located at a much higher value of 853.0 eV, suggesting that the stronger interaction between the Ni species and support. It is further verified by the higher binding energy's location of Ni^2+^ peaks at 853.6 eV and 856 eV. The stronger interaction may endow more electrons transfer to ZrO_2_ support from nickel during the CO_2_ methanation process, thus the Ni 2p peaks of used 50Ni/ZrO_2_ shift to higher binding energies. The two peaks of Ni 2p1/2 are located at 873.6 and 871.2 eV corresponding to Ni^2+^ which with a spin‐energy separation of the Ni 2p_3/2_ and Ni 2p_1/2_ ≈17.6.^[^ ^42^, ^43^, ^44^, ^45^ ^]^ Figure 4d depicts O 1s XPS spectra, which were deconvoluted into three peaks. The peak at the lower binding energy (529.8 eV) was related to lattice oxygen of ZrO_2_ (O_lattice_: O^2–^), the peak at the binding energy (531.4 and 530.7 eV) corresponded to surface oxygen (O_surface_: O^2–^, O_2_^2–^, or O^–^).^[^ ^46^, ^49^, ^51^ ^]^ As for 50Ni/C‐ZrO_2_, the peak at the lower binding energy (529.8 eV) was related to lattice oxygen of ZrO_2_ (O_lattice_: O^2–^), the peak at the binding energy (531.9 and 531.0 eV) corresponded to surface oxygen (O_surface_: O^2–^, O_2_^2–^, or O^–^). There are three kinds of oxygen species over the Ni‐based catalysts. The peaks at 529.8, 531.0, and 532.0 eV over 50Ni/ZrO_2_ are deemed as the lattice oxygen (O_Latt_), surface adsorption oxygen species at OVs, and the adsorbed H_2_O molecule (H_2_O), respectively.^[^ ^46^ ^]^ Upon treating 50Ni/ZrO_2_ under light conditions, it is found that the banding energy of O 1s in 50Ni/ZrO_2_‐used‐5 h displays a positive shift after the reaction, which dues to the generation of more vacancies^[^ ^50^, ^51^, ^52^, ^53^ ^]^ and this result is consistent with the EPR data (Figure 4e). The Zr 3d profiles of 50Ni/C‐ZrO_2_ (Figure S8, Supporting Information) show a main peak of Zr 3d_5/2_ at 181.9 eV and a shoulder peak of Zr 3d_3/2_ at 184.2 eV. It can be obviously observed that the binding energies of 50Ni/ZrO_2_ and 50Ni/ZrO_2_ are used toward higher values (182.1 eV of Zr 3d_5/2_ and 184.4 eV of Zr 3d_3/2_). The binding energies of 3d_5/2_ over 50Ni/C‐ZrO_2_ and 50Ni/ZrO_2_ are lower than the pure stoichiometric ZrO_2_ (182.6 eV), indicating the potential presence of OVs.^[^ ^39^, ^47^, ^48^ ^]^ We further determine the presence and concentration of OVs by employing the electron paramagnetic resonance (EPR) experiments at −173 °C. As displayed in Figure 4e, 50Ni/ZrO_2_ shows characteristic oxygen vacancy signals at *g* = 2.003, which is much stronger than that of 50Ni/C‐ZrO_2_. In addition, 50Ni/ZrO_2_‐used shows a similar strength of OVs signal as the fresh 50Ni/ZrO_2_. The results suggest more OVs exist on the MOF‐derived catalyst 50Ni/ZrO_2_ to favor the CO_2_ conversion, and OVs can maintain stability on 50Ni/ZrO_2_ during the photothermal CO_2_ methanation process. OVs are considered to facilitate the adsorption and activation of CO_2_ during CO_2_ methanation, thus promoting further reaction.^[^ ^54^, ^55^ ^]^

**Figure 5:**
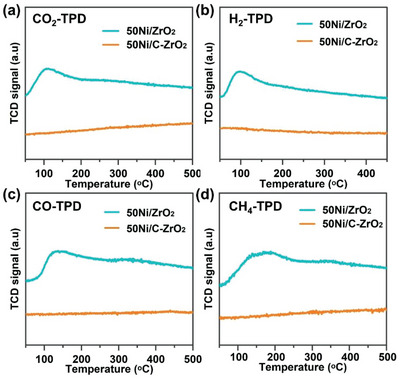
a) CO_2_‐TPD, b) H_2_‐TPD, c) CO‐TPD, d) CH_4_‐TPD profiles of 50Ni/ZrO_2_ and 50Ni/C‐ZrO_2_.

The better adsorption ability of the reactant is beneficial to activate more reactant molecules to participate in the reaction, and the better desorption ability of the product can accelerate the departure of product molecules and further provide more reactive sites to keep the reaction going. In this case, CO_2_‐TPD profiles are performed and shown in **Figure** **5**a. 50Ni/ZrO_2_ shows the intense signal centered ≈100–150 °C, which is ascribed to weak basic sites related to the OH‐groups of the zirconia surface. Moreover, due to the existence of strong basic sites, the signal of CO_2_ desorption extends to 500 °C. However, no peak is observed in 50Ni/C‐ZrO_2_, indicating the poor CO_2_ adsorption ability. A similar phenomenon occurs in the H_2_‐TPD profiles (Figure 5b) that 50Ni/ZrO_2_ shows a stronger peak than 50Ni/C‐ZrO_2_. The results indicate that 50Ni/ZrO_2_ exhibits better adsorption and activation abilities for reactants (CO_2_ and H_2_) to benefit CO_2_ methanation reaction. CO is a crucial intermediate and deserves to be probed for the adsorption behavior during the CO_2_ methanation reaction. As depicted in Figure 5c, 50Ni/ZrO_2_ displays the higher adsorption property of CO compared with 50Ni/C‐ZrO_2_. It is conducive to the hydrogenation reaction and further increases the CH_4_ selectivity, thus accounting for the higher CH_4_ selectivity for 50Ni/ZrO_2_ in light‐driven photothermal catalysis. Furthermore, the CH_4_‐TPD profiles (Figure 5d) show CH_4_ is basically desorbed at 400 °C approaching the temperature during the light‐driven CO_2_ methanation (394 °C), which ensures continuous reaction. According to the analysis above, it can be speculated that MOF‐derived 50Ni/ZrO_2_ generates more tetragonal ZrO_2_ (confirmed by XRD patterns in Figure 1a), which simultaneously strengthens the interaction between nickel and ZrO_2_ and creates more OVs. Hence, 50Ni/ZrO_2_ exhibits enhanced light absorption ability, light‐to‐heat conversion, and superior adsorption capacities of H_2_ and CO_2_ and finally boosts the catalytic performance of the light‐driven CO_2_ methanation.

**Figure 6:**
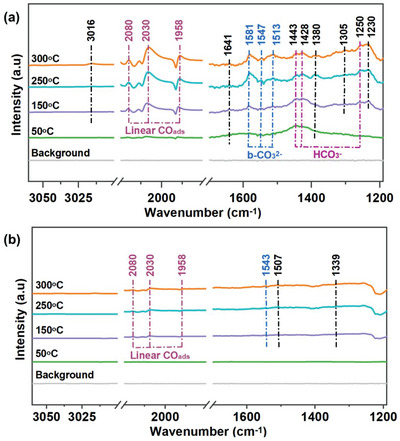
In situ DRIFTS spectra of a) 50Ni/ZrO_2_ and b) 50Ni/C‐ZrO_2_ were measured under light radiation with external heating. During temperature‐programmed reaction: a gas mixture (20 mL min^−1^) containing 10%CO_2_/40%H_2_/50%He was employed.

In situ DRIFTS under the continuous flow of H_2_/CO_2_ gas mixture was performed to identify the involved intermediates and determine the CO_2_ reaction pathways. The intermediates appear in the IR fingerprint regions of 1200–1700, 1900–2500, and 3000–3050 cm^−1^, and the band assignments of surface‐activated species are listed in Table S2 (Supporting Information). For 50Ni/ZrO_2_ in **Figure** **6**a, The bands of bidentate carbonate (b‐CO_3_^2−^, 1581, 1542, and 1513 cm^−1^) and bicarbonate (HCO_3_^−^, 1250, 1428, and 1443 cm^−1^) are observed after the CO_2_/H_2_ mixture gas introduced at low temperature.^[^ ^56^ ^]^ With the temperature gradually rising to 250 °C, the signals of significant intermediates for CH_4_ formation enhance at the bands of 1230, 1380, 1641, and 1900–2100 cm^−1^, which are assigned to COOH*, δ(CH), CH_3_O^−`^, and linear CO species, respectively. When the temperature further rises to 300 °C, the characteristic bands of CH_4_ are detected located at 1305 and 3016 cm^−1^. Simultaneously, similar peaks are observed over the 50Ni/ZrO_2_ without illumination, as shown in (Figure S9, Supporting Information). It suggests that light‐generated electron plays little role in the reaction pathway in the CO_2_ methanation process. As for 50Ni/C‐ZrO_2_ (Figure 6b), the DRIFTS spectra at low temperatures show no adsorption band related to the intermediate product of CO_2_ conversion. The result is consistent with CO_2_‐TPD profiles that 50Ni/ZrO_2_ exhibits superior adsorption capacity for CO_2_ than 50Ni/C‐ZrO_2_. After increasing the reaction temperature, the bands of CO species can be observed, and the peak credited to HCO_3_^−^, m‐CO_3_^2−^, and HCOO* occur at 1543, 1507, and 1339 cm^−1^, respectively. Nevertheless, no key intermediates that are conducive to CH_4_ products are detected. It infers that 50Ni/C‐ZrO_2_ prefers to generate CO during the CO_2_ hydrogenation reaction, which is in accordance with its catalytic performance.

As for 50Ni/Al_2_O_3_, 50Ni/P25, and 50Ni/ZrO_2_‐P (Figure S9, Supporting Information), There are obvious differences in the DRIFTS spectra compared with 50Ni/ZrO_2_, especially in the areas marked in red. It can be observed that 50Ni/ZrO_2_ has more abundant intermediate products and more obvious changes with the increase in reaction temperature. Therefore, it can be reasonably inferred that the 50Ni/ZrO_2_ catalyst is more efficient and the CO_2_ methanation reaction is more active. Hence, the 50Ni/ZrO_2_ catalyst enjoys an advantage over the control samples in the CO_2_ hydrogenation reaction. At the same time, in order to further verify the accuracy and reliability of the conclusion, we conducted XRD characterization and activity evaluation of the three control samples under the Same lighting conditions as 50Ni/ZrO_2_. Finally (Figure S10， S11, Supporting Information), the *r*_CH4_ and *S*_CH4_ of 50Ni/P25 (6.3 mmol g^−1^ h^−1^, 12.5%), 50Ni/Al_2_O_3_ (89.8 mmol g^−1^ h^−1^, 65.5%) and 50Ni/ZrO_2_‐P (201.4 mmol g^−1^ h^−1^, 90.9%) are well below 50Ni/ZrO_2_ during the 2 h photothermal reaction. The above results further prove that 50Ni/ZrO_2_ catalyst is superior in the CO_2_ hydrogenation reaction.

In our work, 50Ni/ZrO_2_ derived from UiO‐66 has higher catalytic performance than 50Ni/C‐ZrO_2_, which may be due to a combination of large surface area, pore volume, and oxygen vacancies. First, the large S_BET_ and total pore volume of UiO‐66 help to be conducive to the homogeneous dispersion of Ni ions and the adsorption of reaction gases to promote CO_2_ methanation reaction. By the way, this pore structure of UiO‐66 may restrict the growth of Ni particles and avoid their agglomeration due to the limiting effect during the calcination process. Moreover, MOFs possess inherited structural properties, which are attractive to 50Ni/ZrO_2_ and form a larger *S*_BET_, *D*_BJH_, and total pore volume to realize a better absorption capacity of light and efficient conversion of CO_2_. Second, oxygen vacancies play an important role in the methanation process. OVs are believed to facilitate CO_2_ adsorption and activation during CO_2_ methanation, resulting in the formation of surface carbon species that undergo methanation reactions with atomic hydrogen, in agreement with the EPR results.^[^ ^59^ ^]^

**Scheme 1:**
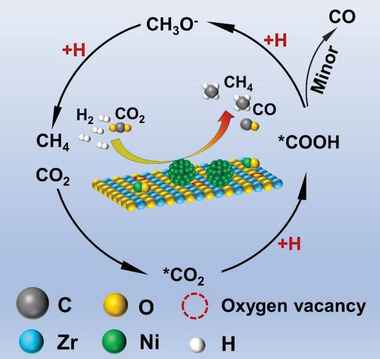
CO and CH_4_ formation mechanism.

Based on the above investigation and published literatures, the formation of CH_4_ in this work follows the formate‐mediated pathway: CO_2_ → *CO_2_ → CO_3_^2−^/HCO_3_^2‐^ → COOH* → *CO → CH_3_O^−^ → CH_4_, and CO is produced by the pathway: CO_2_ → COOH* → CO.^[^ ^56^, ^57^, ^58^, ^59^, ^60^, ^61^, ^62^ ^]^ A description of the associated reaction path is presented in **Scheme** **1**. First, the CO_2_ molecules are adsorbed in the abundant surface OVs and subsequently react with surface hydroxyl or surface oxygen to form activated carbonate species, which next react with atomic hydrogen decomposed from H_2_ and generate COOH*. And then the hydroxyl radical (−OH) is removed from COOH* to generate *CO and H_2_O. For 50Ni/ZrO_2_, a minor amount of the formative *CO is released and the major further participates in the transformation to the methoxy group and finally generates CH_4_.^[^ ^63^ ^]^

## 3 Conclusion

In conclusion, a series of nickel‐based catalysts derived from MOFs have been prepared for photothermal CO_2_ methanation under whole solar light. The optimal catalyst of 50Ni/ZrO_2_ exhibits a CH_4_ yield rate of 583.3 mmol g^−1^ h^−1^, which is ≈10 times higher than that of 50Ni/C‐ZrO_2_ under identical irradiation and exceeds any other Ni‐based catalysts in photothermal CO_2_ methanation. It was found that the MOFs‐derived ZrO_2_ plays a key role in the catalytic performance compared with the commercial ZrO_2_ during the reaction. In addition, the high loading of Ni NPs, effective light‐to‐heat conversion, and more OVs jointly promote the CO_2_ methanation reaction. The in situ DRIFTS show the formation of CO_3_^2−^/HCO_3_^2−^, COOH*, and *CO intermediates, which are the key steps of CO_2_ methanation and indicate the possible reaction pathways over 50Ni/ZrO_2_. This work provides a promising strategy to prepare MOFs‐derived catalysts for high‐efficiency photothermal catalytic CO_2_ methanation. Moreover, we proposed a potentially valuable strategy for photothermal catalytic CO_2_ methanation with strong light‐to‐heat conversion ability.

## 4 Experimental Section

### Materials

All chemicals were analytical grade and obtained from commercial sources without further purification. Commercial zirconium dioxide (ZrO_2_) was bought from Shanghai Macklin Biochemical Co. Ltd. Zirconium chloride (ZrCl_4_) and commercial P25 was obtained from Shanghai Aladdin Biochemical Technology Co., Ltd. Aluminum oxide (γ‐Al_2_O_3_) was purchased from Sigma–Aldrich. Terephthalic acid (H_2_BDC, C_8_H_6_O_4_), N, N dimethylformamide (DMF, C_3_H_7_NO), acetic acid (CH_3_COOH), nickel nitrate hexahydrate (Ni(NO_3_)_2_∙6H_2_O), ammonia solution (NH_3_·H_2_O), and methanol (CH_3_OH) were purchased from Sinopharm Chemical Reagent Co., Ltd. Ultrapure water was obtained from the Millipore Milli‐Q with a resistivity of 18.2 mω cm.

### Catalyst Preparation

UiO‐66 was synthesized by the hydrothermal method with a slight modification from the previous works.^[^ ^31^, ^32^ ^]^ Typically, ZrCl_4_ (233.0 mg) and H_2_BDC (166.1 mg) were dissolved by DMF (50 mL) in a Teflon‐lined autoclave (100 mL). Then, CH_3_COOH (3.6 mL) was added drop by drop and stirred for 30 min to form a homogeneous solution. Next, the autoclave was sealed and heated at 120 °C for 24 h statically. After cooling down to room temperature, the precipitate of UiO‐66 was obtained by centrifugation, rinsed with methanol several times, and finally dried at 100 °C for 12 h.

ZrO_2_‐P was synthesized by the simple co‐precipitation method with a slight modification from the previous works.^[^ ^33^, ^34^ ^]^ Solutions of ZrCl_4_ (1.0 mol L^−1^) were stirred well, and then heated at 60 °C for 30 min. The solution was dropped into 50 mL of 3.0 mol L^−1^ NH_4_OH solution located in a magnetic Stirrer at 60 °C. After 30 min of the reaction, the solution cooled to room temperature, and the resulting white precipitate was then aged for 1.5 h. The aged precipitates were collected by centrifugation and washed with DI water to a neutral pH. The resulting product was oven‐dried at 100 °C overnight and subsequently calcined at 500 °C for 5 h.

The Ni/ZrO_2_ composites were synthesized via the wet impregnation method. First, UiO‐66 powder (200 mg) and a certain amount of Ni(NO_3_)_2_⋅6H_2_O were dispersed into deionized water (100 mL) by ultrasound at room temperature for 10 min. The resulting solution was stirred with a rotation speed of 350 r min^−1^ at room temperature for 6 h and then stirred at 90 °C until dry. The obtained solids were dried at 80 °C overnight. Subsequently, the product powder was calcined in the air atmosphere with the flow of 100 mL min^−1^ at 450 °C for 10 h, and then reduced at 400 °C for 2 h in H_2_ flow (30 mL min^−1^). The as‐obtained products were denoted as xNi/ZrO_2_ (*x* represents the weight percentage of Ni content in the catalyst). The reference sample of 50Ni/C‐ZrO_2_ 50Ni/Al_2_O_3_, 50Ni/P25, 50Ni/ZrO_2_‐P was prepared by the similar procedure except for utilizing commercial ZrO_2_, γ‐Al_2_O_3_, P25, ZrO_2_‐P instead of ZrO_2_ as the support.

### Characterization Methods

The content of Ni in samples was determined via inductively coupled plasma‐optical emission spectrometry (ICP‐OES). The characteristics of samples were analyzed via X‐ray diffraction (XRD) with an X'Pert Pro automatic powder diffractometer utilizing Cu Kα monochromatized radiation (40 kV, 40 mA). The high‐resolution transmission electron microscope (HRTEM) images were obtained from a JEM 2100 F electronic microscopy. The element mapping images of samples were recorded via utilizing energy dispersive spectroscopy (EDS) equipped on the TEM. Nitrogen adsorption‐desorption measurements were carried out on a Quantachrome Autosorb IQ instrument to analyze the average diameter (*D*_BJH_) and specific surface area (*S*_BET_) of samples via the Barrett–Joyner–Halenda (BJH) and Brunauer–Emmett–Teller (BET) methods, respectively. The chemical property of catalysts was detected through a Kratos/Shimadzu X‐ray photoelectron spectroscopy (XPS) with Al Kα radiation (1486.6 eV). The diffuse reflectance spectrum (DRS) was recorded using a Varian Cary 5000 ultraviolet‐visible spectrophotometer which used the standard BaSO_4_ as a reference. Electron paramagnetic resonance (EPR) spectroscopy was recorded on a Bruker A300 spectrometer at the liquid nitrogen temperature, and 10 mg of sample powder was put into the sample tube for testing. The hydrogen temperature‐programmed reduction (H_2_‐TPR), CO_2_ temperature‐programmed desorption (CO_2_‐TPD), H_2_ temperature‐programmed desorption (H_2_‐TPD), and CO pulse adsorption tests were performed on the Quantachrome chemisorption instrument. The detailed process of H_2_‐TPD is as follows. First, 50 mg of sample was pretreated by 5% H_2_/Ar mixture (30 mL min^−1^) at 400 °C for 30 min, and then the gas was switched to Ar flow (30 mL min^−1^) at 400 °C for another 30 min. After the sample was cooled down to 40 °C, 5% H_2_/Ar flow (30 mL min^−1^) was introduced adsorption for 1 h to ensure completely be absorbed into the powder, and then was purged with Ar (30 mL min^−1^ for 1 h) to remove physically adsorbed hydrogen. Finally, H_2_‐TPD was performed under an Ar atmosphere with a heating rate of 10 °C from 40 to 500 °C. The desorbed hydrogen was detected by the thermal conductivity detector (TCD). The CH_4_‐TPD, CO‐TPD, and CO_2_‐TPD were performed using a similar procedure (first in situ reduced by 5% H_2_/Ar at 400 °C, then cooling down by He gas flow), wherein CH_4_, CO, and CO_2_ were introduced as adsorption gases, respectively. H_2_‐TPR was tested as follows: 50 mg of sample was first placed in Ar flow (30 mL min^−1^) at 400 °C for 30 min, and the reduction process was carried out from 25 °C to 600 °C in H_2_ and finally kept at 600 °C for 30 min. The H_2_ electric current signals were tested by TCD.

### In Situ DRIFTs Analysis

In situ DRIFTs were performed under light or heating conditions to investigate the intermediates on a Thermo Fisher iS50 spectrometer with an MCT/A detector. Before the experiments, 50Ni/ZrO_2_ and 50Ni/C‐ZrO_2_ were pretreated in 5%H_2_/Ar for 30 min, then cooled down and collected the spectra as the background at 25 °C in N_2_ flow. Next, 10%CO_2_/40%H_2_/50%He gas mixture was filled into the reaction cell for 15 min. When the mixed gas was fully adsorbed over the sample, the gas mixture was turned off and the tail gas was analyzed by a gas chromatography‐mass spectrometry system (Aglient GC7890‐MS5975C).

### Catalytic Activity Evaluation

The experiment of photothermal CO_2_ methanation was evaluated in an enclosed cylindrical stainless‐steel reactor with a quartz window. A 300 W xenon lamp (PLS‐SXE300UV, Beijing Perfectlight Technology Co., Ltd.,) was employed as a light source for CO_2_ methanation, and light intensity was measured via an optical power meter (CELNP2000‐2, Perfect Light) at the same place of the catalyst layer. The surface temperature of the catalyst layer under light illumination was measured by a thermocouple. Before the catalytic valuation, 10 mg of catalyst powder was flat out on the surface of quartz cotton in the reactor. Before irradiation, the mixed gas of 10%CO_2_/40%H_2_/50%He was introduced into the stainless‐steel reactor for 15 min to remove the air. Then, the xenon lamp was turned on to start the catalytic reaction with the continuous introduced gas mixture (25 mL min^−1^). The UV–vis light or infrared light was obtained through the cut‐off filter to verify different light functions during the photothermal CO_2_ methanation. As a comparison, thermal catalytic activity was measured in a tube furnace. Specifically, 10 mg of the catalyst powder mixed with 1 g of silica sand (sized as 40−60 mesh) was placed into the furnace. The concentration of CO_2_, CO, and CH_4_ was determined by an online GC equipped with a flame ionization detector (FID) and a thermal conductivity detector (TCD). The catalytic durability test for CO_2_ methanation was performed under the same irradiation condition for 10 h. The production rate of CH_4_ (*r*_CH4_) and CO (*r*_CO_) were calculated individually based on the following Equations (1 and 2):

(1) $$r_{\mathrm{CH}4}=\frac{n_{\mathrm{CH}4}}{weight\;of\;catalyst}\;\;\times \;60$$

(2) $$r_{\mathrm{CO}}=\frac{n_{\mathrm{CO}}}{weight\;of\;catalyst}\;\;\times \;60$$

Where,

(3) $$n_{\mathrm{CH}4}=\frac{F\;\times \;\left[{\mathrm{CH}}_{4}\right]}{22.4}$$

(4) $$n_{\mathrm{CO}}=\frac{F\;\times \;\left[\mathrm{CO}\right]}{22.4}$$

Here, *F* represents the gas flow rate (mL min^−1^); [CH_4_] and [CO] are the respective concentrations (vol.%) of CH_4_ and CO detected by online GC.

## Conflict of Interest

The authors declare no conflict of interest.

## Supporting information

Supporting Information

## Data Availability

The data that support the findings of this study are available from the corresponding author upon reasonable request.
