# Supplementary material for: Efficient Solar‐Driven CO2 Methanation and Hydrogen Storage Over Nickel Catalyst Derived from Metal–Organic Frameworks with Rich Oxygen Vacancies
Source: Adv Sci (Weinh). 2023 Oct 22;10(34):2304406. doi: 10.1002/advs.202304406 (PMC10700172; doi:10.1002/advs.202304406)
Supplement: Supplementary file 1 — Supporting Information [file ADVS-10-2304406-s001.pdf]

ADVANCED SCIENCE
Open Access

Supporting Information

for *Adv. Sci.*, DOI 10.1002/advs.202304406

Efficient Solar-Driven CO_2 Methanation and Hydrogen Storage Over Nickel Catalyst Derived from Metal–Organic Frameworks with Rich Oxygen Vacancies

Huiling Wang, Qiang Li, Jin Chen, Jing Chen and Hongpeng Jia**

Appendix A. Supplementary data

Efficient Solar-driven CO_2 Methanation and Hydrogen Storage over Nickel Catalyst Derived from Metal-organic Frameworks with Rich Oxygen Vacancies

Huiling Wang, Qiang Li, Jin Chen, Jing Chen, and Hongpeng Jia**

H. Wang, Q. Li, J. Chen, H. Jia

Xiamen Key Laboratory of Materials for Gaseous Pollutant Control
Institute of Urban Environment
Chinese Academy of Sciences, Xiamen 361021, China
E-mail addresses: jing.chen@fjirsm.ac.cn & hpjia@iue.ac.cn
Tel: 86-592-6190767; Fax: 86-592-6190767

H. Wang, Q. Li, J. Chen, H. Jia

Key Laboratory of Urban Pollutant Conversion
Institute of Urban Environment
Chinese Academy of Sciences
Xiamen 361021, China

H. Wang

College of Life Science
Fujian Agriculture and Forestry University
Fuzhou 350002, China

J. Chen

Fujian Institute of Research on The Structure of Matter
Chinese Academy of Sciences
Fuzhou 350002, China

Q. Li, J. Chen, J, Chen, H. Jia

University of Chinese Academy of Sciences
Beijing 100049, China

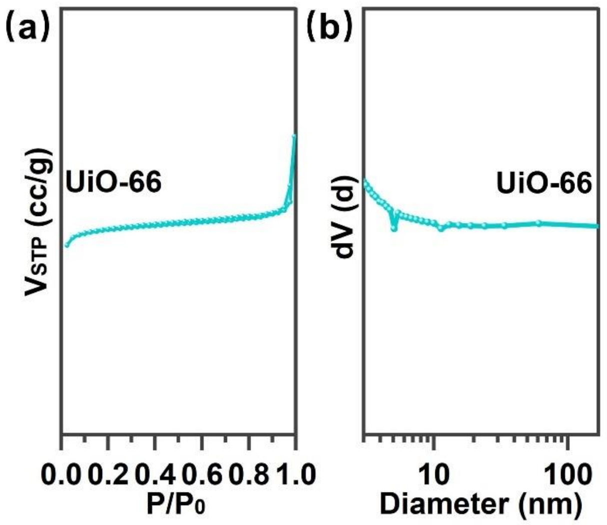

Figure S1. N_2 adsorption-desorption isotherms.

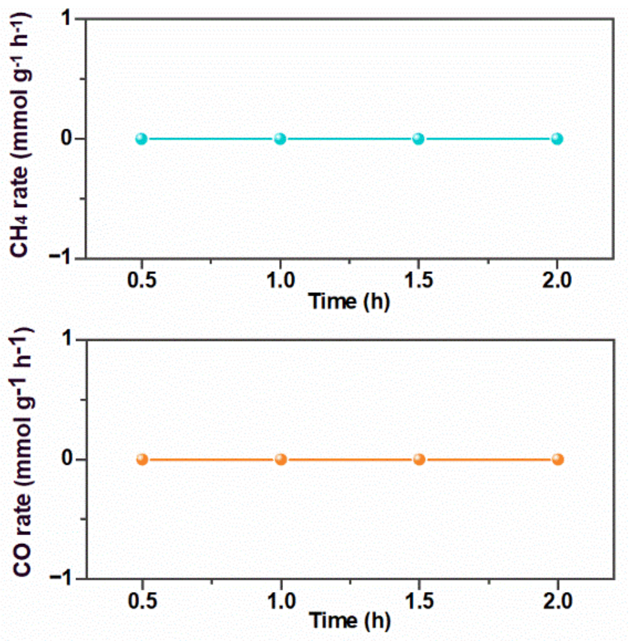

Figure S2. Production rate of ZrO_2 under light irradiation. Reaction conditions: the samples were irradiated under full-spectrum irradiation with light intensities (2.9 W cm^{-2}) with a continuous flow of 10 vol% CO_2, 40 vol% H_2 and 50 vol% He (25 mL min^{-1}).

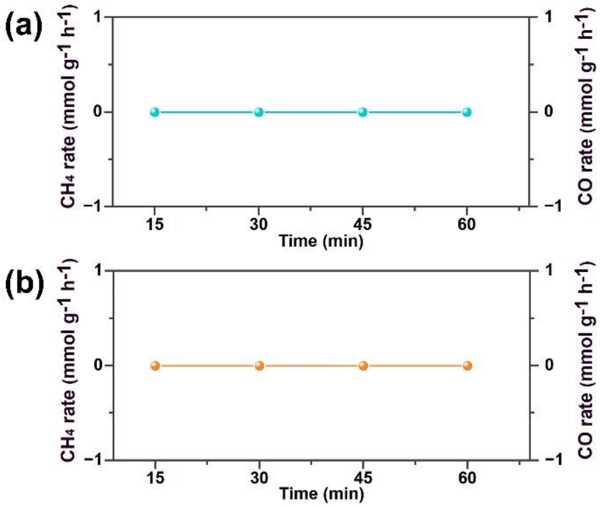

Figure S3. Production rate in the initial 1 h of a) 50Ni/ZrO_2 and b) 50Ni/C-ZrO_2 under 5%H_2/Ar and light intensity of 2.9 W cm^{-2} condition.

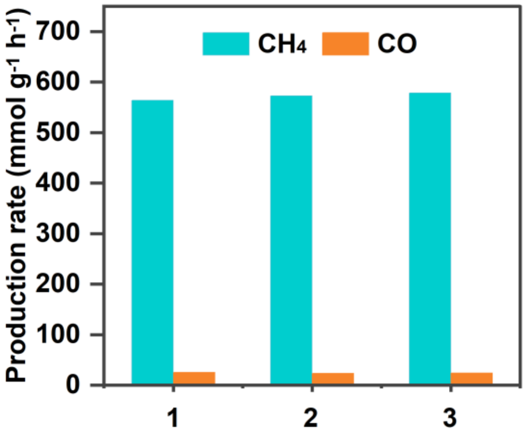

Figure S4. Production rate in the initial 2 h of Three parallel 50Ni/ZrO_2 samples under light intensity of 2.9 W cm^{-2}.

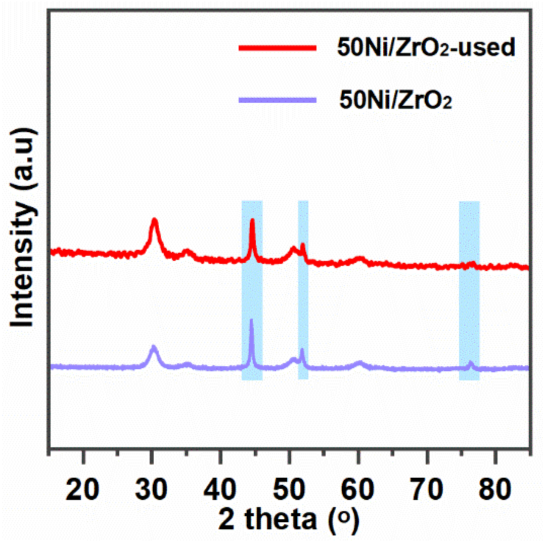

Figure S5. XRD patterns of fresh and used 50Ni/ZrO_2.

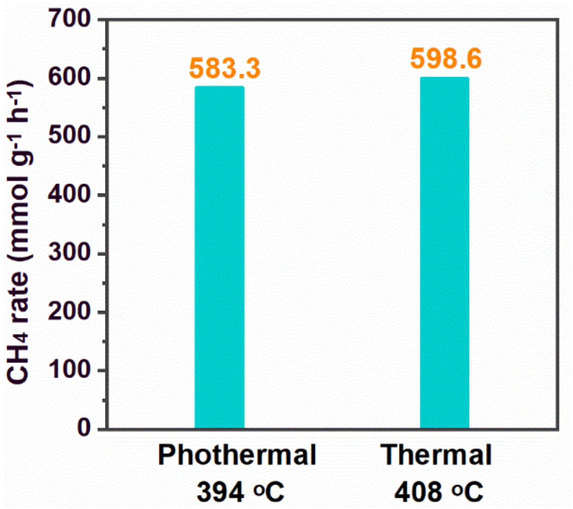

Figure S6. CH_4 rate of 50Ni/ZrO_2 under electric hearting condition.

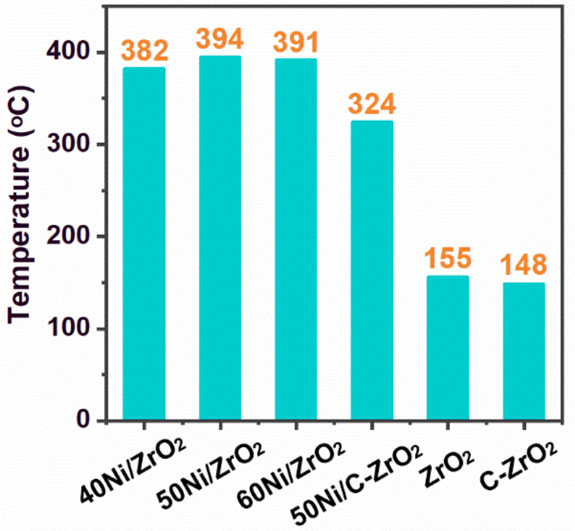

Figure S7. The temperature variation of difference samples under full-spectrum irradiation with light intensities (2.9 W cm^{-2}) for 20 min.

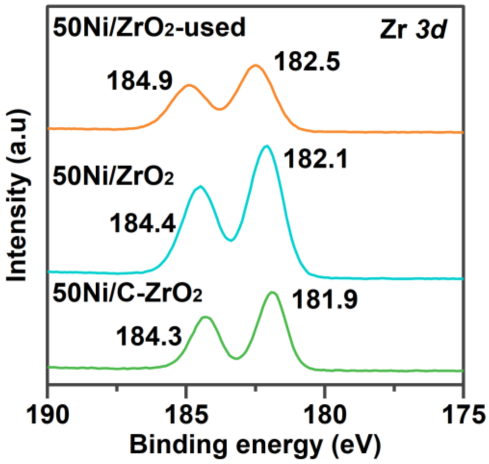

Figure S8. High-resolution XPS data of Zr 3d collected from as-synthesized 50Ni/ZrO_2, 50Ni/C-ZrO_2, and 50Ni/ZrO_2-used.

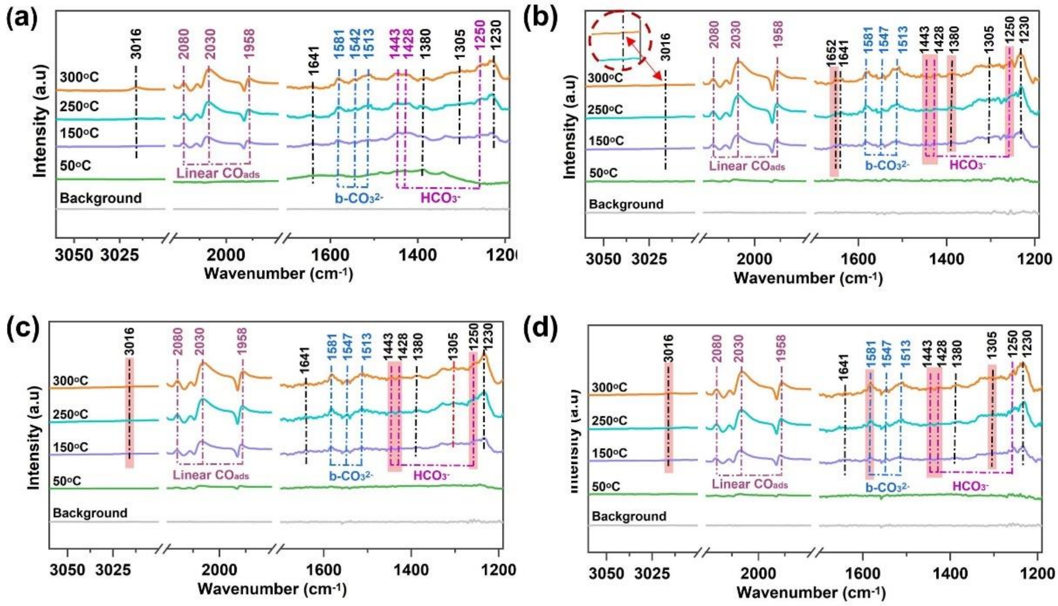

Figure S9. In situ DRIFTS spectra of a) 50Ni/ZrO_2 without light irradiation, b) 50Ni/ZrO_2-P, **c)** 50Ni/P25 and d) 50Ni/Al_2O_3 were measured under light radiation with external heating. During temperature-programmed reaction: a gas mixture (20 mL min^{-1}) containing 10%CO_2/40%H_2/50%He were employed.

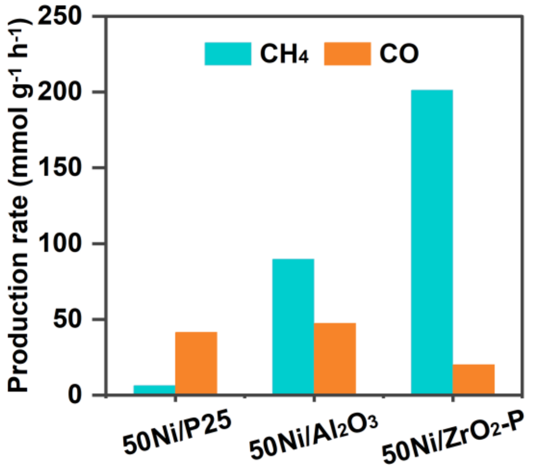

Figure S10. Production rate in the initial 2 h of 50Ni/Al_2O_3, 50Ni/ZrO_2-P, 50Ni/P25 under light intensity of 2.9 W cm^{-2}.

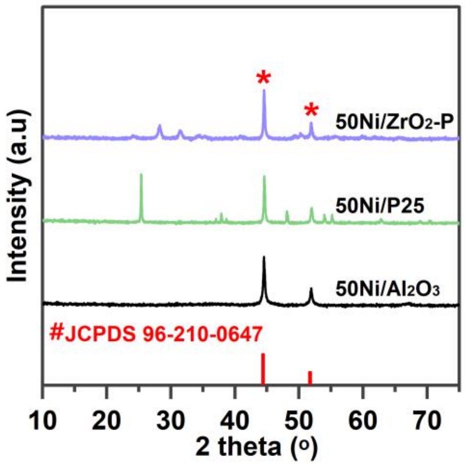

Figure S11. XRD patterns of 50Ni/Al_2O_3, 50Ni/ZrO_2-P, and 50Ni/P25.

Table S1 Catalytic activity for CH_4 production via photo-thermal route in the reported literature.

Catalysts	Surface temperature (^oC)	CH_4 production (mmol $g_{cat}^{-1}h^{-1}$)	Ref
50Ni/ZrO_2	394	583.3	This work
8Ni/TiO_2	390	37.1	[1]
Ni/$BaTiO_3$	390	103.7^a	[2]
Ni/Al_2O_3	396	25.0	[3]
Ru/MnO-$MgCO_3$	400	50.7	[4]
Rh/γ-Al_2O_3	373	379	[5]
Ni/CeO_2–IM	450	26.4	[6]
NiCe-1.00	400	214	[7]
8.68% Ni/ZrO_2	350	325.8	[8]
10 wt% Ni/CeO_2	340	82.1	[9]
Rh/Al	700	240	[10]
Ni@C-600	320	488	[11]
0.16%Ir-CoO/Al_2O_3	250	128.9	[12]
Ru-TiOx	276	15.84	[13]
NiAl-LDH/CeO_2-20	-	0.016	[14]
Ni/BN-2	230℃+light	2028.5^b	[15]
Ru/HNT	327	1704	[16]
0.35%Ru@$Ni_2V_2O_7$	350	114.9	[17]
Au/meso-Co_3O_4	350	204	[17]

$^{a)}$ The experiment was carried out under 5 bar pressure.

$^{b)}$ The production rate is based on mmol $g_{Ni}^{-1}h^{-1}$.

Table S2 Vibrational wavenumbers measured in this work and collected from the literature.

Position (cm^{-1})	Corresponding species	reference
1230, 1590	COOH*	[18, 19]
1341	$*CH_3$	[20]
1380	δ(CH)	[21]
1250, 1442, 1443	HCO_3^-	[17, 22, 23]
1507	m-CO_3^{2-}	[24]
1513, 1542, 1581	b-CO_3^{2-}	[25]
1641	CH_3O^-	[26]
1900-2100	CO	[8, 22]
2341, 2362	CO_2	[27]
1305, 3016	CH_4	[18]

References

[1] Q. Li, Y. Gao, M. Zhang, H. Gao, J. Chen, H. Jia, *Appl. Catal. B*. **2022**, 303, 120905.

[2] D. Mateo, N. Morlanes, P. Maity, G. Shterk, O.F. Mohammed, J. Gascon, *Adv. Funct. Mater.* **2020**, 31, 6188-6195.

[3] X. Meng, T. Wang, L. Liu, S. Ouyang, P. Li, H. Hu, T. Kako, H. Iwai, A. Tanaka, J. Ye, *Angew. Chem., Int. Ed*. **2014**, 53, 11478-11482.

[4] Q. Wang, Y. Gao, C. Tumurbaatar, T. Bold, F. Wei, Y. Dai, Y. Yang, *J. Energy Chem*. **2022**, 64, 38-46.

[5] M. Jacquemin, A. Beuls, P. Ruiz, *Catal. Today*. 2010, 157, 462-466.

[6] R.-P. Ye, Q. Li, W. Gong, T. Wang, J.J. Razink, L. Lin, Y.-Y. Qin, Z. Zhou, H. Adidharma, J. Tang, A.G. Russell, M. Fan, Y.-G. Yao, *Appl. Catal. B*. **2020**, 268, 118474.

[7] G. Varvoutis, M. Lykaki, S. Stefa, V. Binas, G.E. Marnellos, M. Konsolakis, *Appl. Catal. B*. **2021**, 297,12401.

[8] X. Jia, X. Zhang, N. Rui, X. Hu, C.-j. Liu, *Appl. Catal. B*. **2019**, 244, 159-169.

[9] A. Löfberg, J. Guerrero-Caballero, T. Kane, A. Rubbens, L. Jalowiecki-Duhamel,

Appl. Catal., B. **2017**, 212, 159-174.

[10] G. Fu, M. Jiang, J. Liu, K. Zhang, Y. Hu, Y. Xiong, A. Tao, Z. Tie, Z. Jin, Rh/Al *Nano Lett*, **2021**, 21, 8824-8830.

[11] I. Khan, D. Mateo, G. Shterk, T. Shoinkhorova, D. Poloneeva, L. Garzón-Tovar, J. Gascon, *Angew. Chem. Int. Ed*. **2021**, 60, 26476-26482.

[12] Y. Tang, T. Zhao, H. Han, Z. Yang, J. Liu, X. Wen, F. Wang, *Adv. Sci*. **2023**, 10, 2300122.

[13] T. Dong, X. Liu, Z. Tang, H. Yuan, D. Jiang, Y. Wang, Z. Liu, X. Zhang, S. Huang, H. Liu, L. Zhao, W. Zhou, *Appl. Catal. B*. **2023**, 326, 122176.

[14] M. Yang, X. Zhu, Z. Zhu, H. Zhang, Y. Teng, D.-B. Kuang, Y. Li, *Chem. Eng. J.* **2023**, 472, 145071.

[15] X. Zhu, H. Zong, C.J.V. Pérez, H. Miao, W. Sun, Z. Yuan, S. Wang, G. Zeng, H. Xu, Z. Jiang, G.A. Ozin, *Angew. Chem. Int. Ed*. **2023**, 62, e202218694.

[16] K. Peng, J. Ye, H. Wang, H. Song, B. Deng, S. Song, Y. Wang, L. Zuo, J. Ye, *Appl. Catal. B*. **2023**, 324, 122262.

[17] Y. Chen, Y. Zhang, G. Fan, L. Song, G. Jia, H. Huang, S. Ouyang, J. Ye, Z. Li, Z. Zou, *Joule*. **2021**, 5, 3235-3251.

[18] X. Zu, Y. Zhao, X. Li, R. Chen, W. Shao, Z. Wang, J. Hu, J. Zhu, Y. Pan, Y. Sun, Y. Xie, Angew. *Chem., Int. Ed*. **2021**, 60, 13840-13846.

[19] Z.-j. Wang, H. Song, H. Pang, Y. Ning, T.D. Dao, Z. Wang, H. Chen, Y. Weng, Q. Fu, T. Nagao, Y. Fang, J. Ye, *Appl. Catal. B*. **2019**, 250, 10-16.

[20] Z. Rao, Y. Cao, Z. Huang, Z. Yin, W. Wan, M. Ma, Y. Wu, J. Wang, G. Yang, Y. Cui, Z. Gong, Y. Zhou, *ACS Catalysis*, **2021**, 11, 4730-4738.

[21] M. Kantcheva, M.U. Kucukkal, S. Suzer, *J. Catal*. **2000**, 190, 144-156.

[22] W.L. Vrijburg, E. Moioli, W. Chen, M. Zhang, B.J.P. Terlingen, B. Zijlstra, I.A.W. Filot, A. Züttel, E.A. Pidko, E.J.M. Hensen, *ACS Catalysis* **2019**, 9, 7823-7839.

[23] M.M. Millet, G. Algara-Siller, S. Wrabetz, A. Mazheika, F. Girgsdies, D. Teschner, F. Seitz, A. Tarasov, S.V. Levchenko, R. Schlogl, E. Frei, *J. Am. Chem. Soc*. **2019**, 141, 2451-2461.

[24] G. Ren, Z. Wei, S. Liu, M. Shi, Z. Li, X. Meng, *Chemosphere* **2022**, 307, 136026.

[25] Y. Yan, Y. Dai, H. He, Y. Yu, Y. Yang, *Appl. Catal. B*. **2016**, 196, 108-116.

[26] S. Cai, M. Zhang, J. Li, J. Chen, H. Jia, *Sol. RRL*. **2020**, 5, 2000313.

[27] Y. Gao, Q. Li, C. Wang, D. Yan, J. Chen, H. Jia,*J. Mater. Chem. A*. **2022**, 10, 16016-16028.
